# Comparative transcriptomics gives insights into the evolution of parasitism in *Strongyloides* nematodes at the genus, subclade and species level

**DOI:** 10.1038/s41598-018-23514-z

**Published:** 2018-03-26

**Authors:** Vicky L. Hunt, Akina Hino, Akemi Yoshida, Taisei Kikuchi

**Affiliations:** 1 Parasitology, Faculty of Medicine, University of Miyazaki, Miyazaki, 889-1692 Japan; 2 Department of Environmental Parasitology, Graduate School of Medical and Dental Sciences, Tokyo Medical and Dental University, 1-5-45 Yushima, Bunkyo-ku, Tokyo, 113-8510 Japan; 3 Genomics and Bioenvironmental Science, Frontier Science Research Center, University of Miyazaki, Miyazaki, 889-1692 Japan

## Abstract

*Strongyloides* spp., gastrointestinal nematode parasites of humans and other animals, have genetically identical parasitic and free-living adult life cycle stages. This is an almost unique feature amongst nematodes and comparison of these two stages can provide insights into the genetic basis and evolution of *Strongyloides* nematode parasitism. Here, we present RNAseq data for *S*. *venezuelensis*, a parasite of rodents, and identify genes that are differentially expressed in parasitic and free-living life cycle stages. Comparison of these data with analogous RNAseq data for three other *Strongyloides* spp., has identified key protein-coding gene families with a putative role in parasitism including WAGO-like Argonautes (at the genus level) and speckle-type POZ-like coding genes (*S*. *venezuelensis-S*. *papillosus* phylogenetic subclade level). Diverse gene families are uniquely upregulated in the parasitic stage of all four *Strongyloides* species, including a distinct upregulation of genes encoding cytochrome P450 in *S*. *venezuelensis*, suggesting some diversification of the molecular tools used in the parasitic life cycle stage among individual species. Together, our results identify key gene families with a putative role in *Strongyloides* parasitism or features of the parasitic life cycle stage, and deepen our understanding of parasitism evolution among *Strongyloides* species.

## Introduction

*Strongyloides* nematodes are soil-transmitted gastrointestinal parasites of humans and other animals. The *Strongyloides* life cycle has genetically identical adult parasitic female (PF) and free-living female (FLF) stages^1^, an almost unique feature amongst nematodes. The PF inhabits the mucosa of the small intestine of its host and produces genetically identical offspring by mitotic parthenogenesis. Eggs leave the host with the faeces and develop into infective larvae stage (iL3), which infect a new host via percutaneous penetration, either directly or via a free-living, dioecious adult generation. The free-living adult generation lives independently of a host and is not parasitic^2^. Because PF and FLF are genetically identical, differences between these two life cycle stages must be attributed to differences in transcriptional or post-transcriptional regulation of their genes. Direct comparisons between the transcriptomes of the PF and FLF can identify differentially expressed genes, enabling inferences to be made about the genes, and the proteins they code for, with a putative role in parasitism, or features associated with the parasitic life style^3^.

The genomes of four *Strongyloides* species – *S*. *ratti*, *S*. *stercoralis*, *S*. *venezuelensis* and *S*. *papillosus* – and two closely related species, *Parastrongyloides trichosuri* (parasitic nematode) and *Rhabditophanes* sp. (free-living nematode) have recently been sequenced^3^. The genome sequences revealed they possess robust gene homology and synteny across species. Comparison of *S*. *ratti* and *S*. *stercoralis* RNAseq data for PF and FLF stages of the life cycle has uncovered key gene families with a putative role in *Strongyloides* parasitism^3,4^. Interestingly, many of the gene and protein families identified as having a putative role in *Strongyloides* parasitism, based on transcriptome and proteome evidence, are also reported to have a role in parasitism in a range of other parasitic nematode species^5–7^. *Strongyloides* spp. can therefore provide a suitable model system for studying the genetic and molecular basis of nematode parasitism, more generally.

The four sequenced *Strongyloides* species can be phylogenetically grouped into two distinct subclades: *S*. *venezuelensis-S*. *papillosus* and *S*. *ratti-S*. *stercoralis*. These subclades can be characterised by the number of chromosomes they possess - *S*. *ratti* and *S*. *stercorali*s have two autosomes and one X chromosome; *S*. *venezuelensis* and *S*. *papillosus* possess two chromosomes (including one chromosome comprising a fusion of *S*. *stercoralis-S*. *ratti* chromosomes I and X), signifying some level of evolutionary divergence between these two subclades^8–10^. Different *Strongyloides* species have evolved to parasitise distinct and varied hosts; *S*. *venezuelensis* and *S*. *ratti* are parasites of rodents, *S*. *stercoralis* is a parasite of humans and dogs, and *S*. *papillosus* is a parasite of sheep. Specialism of *Strongyloides* species to their specific hosts indicates further evolutionary diversification within the *Strongyloides* nematode clade. Comparative analyses of the PF and FLF transcriptomes of these four *Strongyloides* species offers an interesting comparison of genes, and the proteins they code for, involved in parasitism, and can provide insights into the evolution of parasitism at the genus, subclade and species level. Here, we have performed RNAseq analysis of *S*. *venezuelensis* PF and FLF life cycle stages and compared these data with PF and FLF transcriptomes of three other *Strongyloides* species, to identify common and unique gene families putatively important in *Strongyloides* parasitism.

## Results

### Distinct sets of genes are differentially expressed in the parasitic and free-living adult females of *Strongyloides* spp

**Figure 1:**
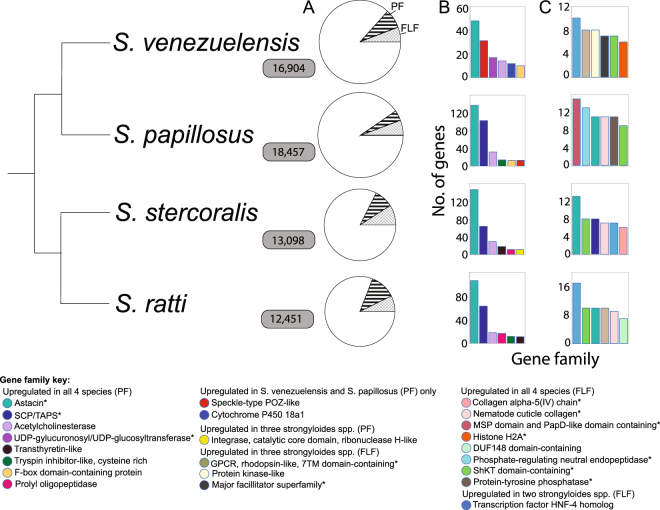
Differentially expressed genes in parasitic and free-living adult female life cycle stages for four *Strongyloides* species – *S*. *venezuelensis*, *S*. *papillosus*, *S*. *ratti* and *S*. *stercoralis*. (**A**) Proportion of genes upregulated in the parasitic and free-living adult female life cycle stages of four *Strongyloides* spp. Total area of the circle is proportional to the total number of predicted protein-coding genes in the genome (total gene number shown in grey boxes). Proportion of genes differentially expressed for a parasitic female (PF) vs. free-living females (FLF) pairwise comparison, determined by edgeR analysis are highlighted (PF – stripes, FLF – squares). (**B**,**C**) The six most commonly upregulated gene families are displayed for the parasitic (**B**) and free-living (**C**) stages of the life cycle n.b. different y-axis scales. *Represents families where distinct sets of genes belonging to the same family are differentially expressed in both life cycle stages.

In total, 1257 and 969 genes were upregulated in the FLF and PF stages of the *S*. *venezuelensis* life cycle, respectively (Fig. 1, Supplementary Table S1**)**. We compared these data to the genes that are differentially expressed across the analogous life cycle stages in three other Strongyloides species: *S*. *papillosus* (for consistency in data analysis we have reanalysed the raw RNAseq data published by Baskaran *et al*.^11^) (Supplementary Table S2), and analysed RNAseq data we have previously published for *S*. *ratti* and *S*. *stercoralis* transcriptomes^3^. The proportion of genes differentially expressed between the PF and FLF stages for all four species ranged between 10–18%, however, the absolute number of differentially expressed genes were similar for three species – *S*. *venezuelensis*, *S*. *stercoralis and S*. *ratti* (2297–2379) and lower for *S*. *papillosus* (1761 genes) (Fig. 1A, Supplementary Fig. S1).

Across all four *Strongyloides* species, the differentially expressed genes belong to 3479 orthologue families (‘orthofamilies’, as defined by Hunt *et al*.^3^), and among them 58 and 105 orthofamilies were commonly upregulated in all four species at the PF and FLF stages, respectively (Supplementary Fig. S2a). Differentially expressed genes were further classified into ‘gene families’ based on the predicted function of the protein they encode^3^ (Supplementary Tables 3–6).

Genes from 29 gene families were commonly upregulated in the PF of all four species, and include families previously identified as having a putative role in parasitism such as acetylcholinesterase, astacin-like metalloendopeptidase (‘astacins’), SCP/TAPS, prolyl endopeptidase, transthyretin-like and trypsin inhibitors^3,4^ (Fig. 1, Supplementary Table S3). The astacins and SCP/TAPS-coding genes dominate the upregulated transcriptome of the PF (*cf*. FLF transcriptome) in *S*. *ratti*, *S*. *stercoralis*^3^ and *S*. *papillosus*^11^. In *S*. *venezuelensis*, astacin-coding genes were also the most commonly upregulated family in the PF transcriptome. However, only 2 SCP/TAPS-coding genes (of 159 SCP/TAPS-coding genes in the genome) were detected as upregulated in the *S*. *venezuelensis* PF transcriptome compared with 63, 64 and 102 genes (of 89, 113 and 205 SCP/TAPS-coding genes in the genome) in the PF transcriptome of *S*. *ratti*, *S*. *stercoralis* and *S*. *papillosus*, respectively. Overall, the expression levels of *S*. *venezuelensis* SCP/TAPS-coding genes were higher in PFs than for FLFs but genes in both life cycle stages had low read numbers (Supplementary Table S7). Interestingly, transcriptome analysis of six *S*. *venezuelensis* life cycle stages show that expression levels of SCP/TAPS-coding genes are higher at the life cycle stages inside the host i.e. the third stage larvae in the lungs (lL3), young parasitic females (yPF) and gravid parasitic females (gPF), compared with life cycle stages external to the host i.e. eggs, first/second stage larvae (L1/2) and free-living infective larvae stages (iL3) (Supplementary Fig. S3). This suggests that SCP/TAPS are important at the larval and adult stages of parasitism inside the host.

A wider range of gene families were upregulated in the FLF transcriptome, including 57 gene families, belonging to 105 orthofamilies, commonly upregulated by FLFs of all four *Strongyloides* species (Supplementary Table S5, Supplementary Fig. S2b). In general, FLF-upregulated gene families comprised fewer genes compared with the gene families upregulated across all four PF transcriptomes (Fig. 1). This pattern is akin to previous observations we have reported for *S*. *ratti* and *S*. *stercoralis* FLF and PF transcriptomes^3^, and indicates the importance of a smaller number of key gene families involved in the parasitic, compared to free-living adult life cycle stage. In some cases, distinct subsets of genes from the same gene families were differentially upregulated across both PF and FLF transcriptomes. For example, genes from ShKT domain-containing proteins, SCP/TAPS, astacins and protein-tyrosine phosphatase gene families were upregulated in both the PF and FLF transcriptomes of all four *Strongyloides* species (Fig. 1), suggesting that some gene families have different roles at different life cycle stages.

**Figure 2:**
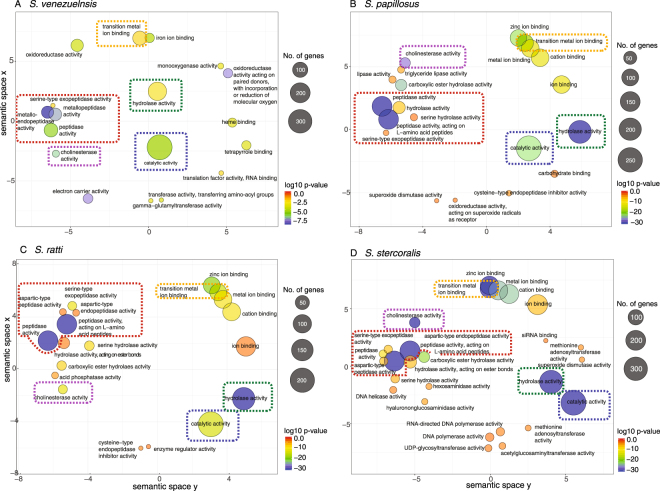
Summary of Molecular Function Gene Ontology (GO) enriched in the parasitic female (PF) stage for four species: (**A**) *S*. *venezuelensis*, (**B**) *S*. *papillosus*, (**C**) *S*. *ratti* and (**D**) *S*. *stercoralis*. GO terms commonly upregulated in the PF of all four species are highlighted by dashed colour-coded boxes including peptidase activity (red), transition metal ion binding (orange), hydrolase activity (green), catalytic activity (blue) and cholinesterase activity (purple). Circle size is proportional to the number of genes associated with each GO term that is significantly upregulated in the PF life cycle stage. Circle colour represents the p-value for GO terms significantly upregulated in PFs. Clustering of GO terms and semantic space x and y are calculated by REVIGO^31^ which uses a clustering algorithm to generate a measurement of semantic similarity.

GO analysis of *S*. *venezuelensis* data revealed 20 Molecular function (MF), 10 Biological Processes (BP) and 6 Cellular components (CC) GO terms were enriched amongst genes upregulated in the PF stage (Fig. 2, Supplementary Fig. S4) and 21 (MF), 55 (BP), 12 (CC) are enriched for upregulated genes in the FLF stage (Supplementary Fig. S4). The overrepresented GO terms of PF and FLF *S*. *venezuelensis* were compared with overrepresented GO terms of *S*. *ratti*, *S*. *stercoralis*^3^ and *S*. *papillosus* PF and FLF to identify commonalities and differences between these four *Strongyloides* species (Supplementary Tables S8–S13). Ten MF GO terms, mostly relating to peptidase activity including metallopeptidase (GO:0004222, GO:0008237), and endopeptidase (GO:0004175) activity were commonly overrepresented in the transcriptome of PF from all four species, highlighting the importance of peptidase activity in *Strongyloides* parasitism. Cholinesterase (GO:0004104) activity is also enriched in the PF stage, supporting previous observations that cholinesterases have a putative role in *Strongyloides* parasitism^4^ (Supplementary Table S3, Supplementary Fig. S4).

### Worm-specific Argonautes (WAGO)-like have a putative role in the *Strongyloides* parasitic stage

**Figure 3:**
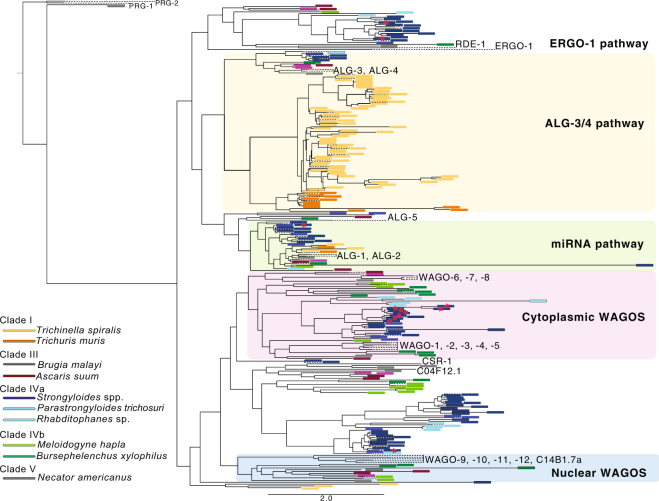
Phylogeny of presumed Argonautes in nematodes. Genes coding for predicted Argonaute proteins from 14 species spanning five evolutionary clades are coloured according to species except for the four *Strongyloides* species (*S*. *venezuelensis*, *S*. *papillosus*, *S*. *ratti* and *S*. *stercoralis*) which are all colour-coded in dark blue. Genes belonging to each species are colour-coded with similar colours according to their evolutionary clade (as defined by Blaxter *et al*.^32^). The protein name of *C*. *elegans* Argonautes is shown directly on the phylogeny (black text), and the Argonaute family that clusters of genes are predicted to belong to, are highlighted according to the most closely related *C*. *elegans* Argonaute family. Genes coding for Argonautes upregulated in the parasitic female, compared with free-living female, life cycle stage of *Strongyloides* are indicated by red stars. *Strongyloides* genes code for Argonautes closely related to most of the Argonautes families defined in *C*. *elegans*, but like other nematodes outside of Clade V, are missing PIWI Argonautes (PRG-1/-2). Scale bar represents average amino acid substitutions per site.

**Figure 4:**
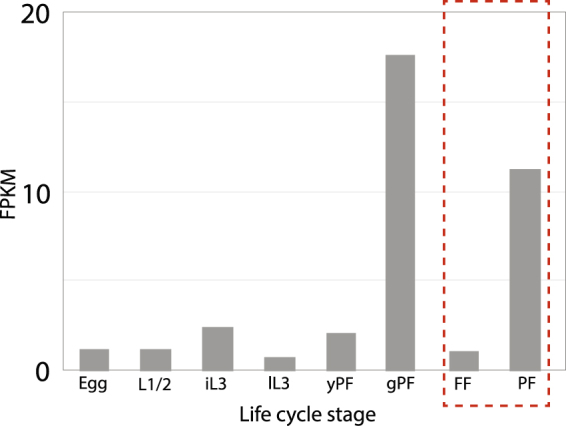
Gene expression of a *S*. *venezuelensis* Cytoplasmic WAGO in each developmental stage. Median expression levels calculated from biological replicates (three replicates for PF and FLF; two replicates for all other life cycle stages) for a WAGO-like coding gene (GenBank ID: LM524968.1) upregulated in the parasitic adult female (PF) compared with free-living adult females (FLF) stage of *S*. *venezuelensis* (red dashed line). Expression levels across six life cycle stages, including eggs, first/second-stage larvae (L1/2), infective third-stage larvae (iL3), infective larvae isolated from the host lung (lL3), young PF (yPF) i.e. without eggs, and gravid PF (gPF) i.e. carrying eggs are shown in the chart. Note, PF and gPF samples are both prepared from gravid parasitic females at similar dpi but the library construction methods (amplified and non-amplified) and sequencing run batches and machines (MiSeq and HiSeq) differ, respectively. FPKM = Fragments Per Kilobase per Million mapped reads.

Between one and six genes encoding Argonaute proteins were upregulated in the PF of all four *Strongyloides* species. Phylogenetic analysis of Argonautes for the four *Strongyloides* spp., two closely related species, *P*. *trichosuri* and *Rhabditophanes* sp., and eight outgroups species spanning four further evolutionary clades revealed that a group of Argonautes comprising genes upregulated in the PF of all four species, were most closely related to the cytoplasmic WAGO family of Argonautes (*C*. *elegans* WAGO-1–5) (Fig. 3, Supplementary Fig. S5). In *C*. *elegans*, WAGOs bind to secondary siRNAs and have a role in amplifying the RNAi signal in the ERGO-1, ALG-3/4 and PIWI pathways, which are typically active in the germline^12,13^. Both the PF and FLF samples analysed here carry eggs and differences in gene expression levels could be associated with the germline cells. To further elucidate the expression pattern of cytoplasmic WAGO-like coding genes we examined the expression level of the WAGO-like coding gene (SVE_0524300.1) upregulated in the PF stage of *S*. *venezuelensis*, across several further life cycle stages including eggs, larval stages, young parasitic adult (i.e. that do not carry eggs) and gravid parasitic adult stages of *S*. *venezuelensis* (Fig. 4). WAGO-like gene expression was greater in gravid PF, compared to young PF and early life cycle stages including eggs and larval stages. From this we can conclude that the *S*. *venezuelensis* WAGO-like Argonaute gene is upregulated in either the (i) PF, specifically at the mature, gravid stage or (ii) matured ovaries or eggs when inside, compared with outside, of the PF.

It is also interesting to note that *Strongyloides* nematodes possess families of Argonautes similar to those of the *C*. *elegans* families, including ERGO-1, ALG-3/4 and ALG-1/2 (microRNA pathway) (Fig. 3). However, the Argonautes most similar to the CSR-1 Argonaute appear to have diversified, and *Strongyloides* Argonautes closely related to nuclear WAGOs were absent. The phylogeny confirms previous findings that Argonautes PRG-1/2 (involved in the PIWI pathway) are absent for nematodes outside of phylogenetic nematode clade V to which *C*. *elegans* belongs^14^, including *Strongyloides* species^15^ (Fig. 3).

### The expanded speckle-type POZ-like gene family is upregulated in parasitic females of the *S. venezuelensis-S. papillosus*, but not *S. ratti-S. stercoralisStrongyloides* subclade

In *S*. *venezuelensis*, 31 genes encoding speckle-type POZ protein-like (SPOP-like) proteins, were upregulated in PF compared with FLF (Fig. 1, Supplementary Table S1). In *S*. *papillosus*, from the same phylogenetic subclade, 13 SPOP-like genes were upregulated in the PF (*cf*. FLF). In comparison, no genes encoding SPOP-like were differentially expressed between PF and FLF for *S*. *ratti* and *S*. *stercoralis*. This suggests that the SPOP-like protein family has a role in parasitism or another feature associated with PF biology for the *S*. *venezuelensis-S*. *papillosus* subclade, but not the *S*. *ratti*-*S*. *stercoralis* subclade of *Strongyloides* nematodes. The SPOP-like family is greatly expanded in *S*. *venezuelensis* (48 genes) and *S*. *papillosus* (61 genes), compared to other nematodes including other *Strongyloides* and *Parastrongyloides* species, which possess between 0–2 SPOP-like encoding genes in their genomes^3^. The SPOP proteins are components of the cullin-RING-based BCR (BTB-CUL3-RBX1) E3 ubiquitin-protein ligase complex, commonly involved in proteasomal degradation of targeted proteins in the ubiquitin proteasome pathway (UPP). Interestingly, a greater number of genes encoding F-box proteins, which play a role in recruiting substrates to a core ubiquitin, are also upregulated in the PF of *S*. *venezuelensis* (10 genes) and *S*. *papillosus* (13 genes), than those upregulated in the PF of *S*. *ratti* (two genes) and *S stercoralis* (four genes). Together these results suggest that ubiquitination is upregulated in the PF of *S*. *venezuelensis* and S. *papillosus*, compared with *S*. *ratti* and *S*. *stercoralis*.

**Figure 5:**
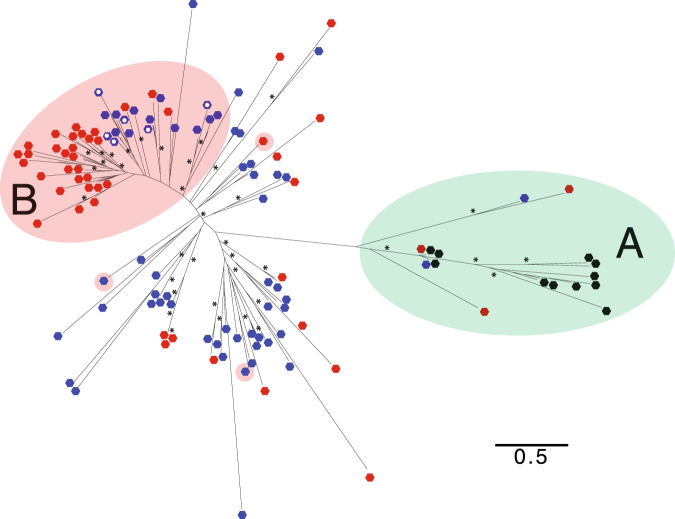
Phylogeny of speckle-type POZ-like (SPOP-like) genes. Amino acid sequences for predicted SPOP-like genes from 14 nematodes species, spanning five evolutionary clades (as defined by Blaxter *et al*.^32^), were used to construct a phylogeny. SPOP-like genes are highlighted for *S*. *venezuelensis* (red) and *S*. *papillosus* (blue); genes for all other species are shown in black. A gene cluster present in all 14 species is shown with a green background highlight (**A**). Genes upregulated in the transcriptome of the parasitic adult female, compared with the free-living adult female, are shown with background highlighting for *S*. *venezuelensis* and *S*. *papillosus* (pink background (**B**), excluding genes shown with a white spot). Scale bar represents average amino acid substitutions per site. *Represents branch support values greater than 70, for 100 bootstraps. Gene names for all genes used to build this phylogenetic tree are shown in Supplementary Fig. S6.

Phylogenetic analysis of 120 SPOP-like protein sequences from 14 nematode species, including *S*. *venezuelensis*, *S*. *papillosus*, *S*. *ratti* and *S*. *stercoralis* show that the expanded SPOP-like gene family in *S*. *venezuelensis* and *S*. *papillosus* is distinct from the SPOP-like genes found in other nematodes (Fig. 5, Supplementary Fig. S6). SPOP-like genes from the genomes of 12 nematodes, excluding *S*. *venezuelensis* and *S*. *papillosus*, formed a single cluster which also included two *S*. *venezuelensis* and two *S*. *papillosus* genes. All of the SPOP-like genes from this cluster were predicted to encode two protein domains - BTB/POZ (IPR000210), a conserved protein-protein interaction domain^16^, and a MATH/TRAF domain (IPR002083), involved in substrate recognition and binding^17^ - except for the four *S*. *venezuelensis* and *S*. *papillosus* genes in this cluster which encoded either one or neither of these domains. Genes in this conserved cluster were expressed, but not differentially so, between the PF and FLF stages. In total, 23* S*. *venezuelensis* and 38 *S*. *papillosus* SPOP-like encoding genes coded for both a BTB/POZ and a MATH/TRAF domain, including 17 and nine genes, respectively, that were upregulated in the PF. Twenty-nine and eleven of the 31 SPOP-like genes upregulated in the PF stage of *S*. *venezuelensis* and *S*. *papillosus*, respectively, formed a distinct phylogenetic cluster, supporting that the expansion of this cluster is important in *S*. *venezuelensis* parasitism (Fig. 5). Together the expression data and SPOP-like phylogeny suggest that the SPOP-like gene family have diversified and the proteins they could code for have possibly adapted novel functions in the *S*. *venezuelensis-S*. *papillosus* subclade compared to other nematodes species including *S*. *ratti* and *S*. *stercoralis*.

### Genetic basis of host specialisation

A diverse range of gene families were uniquely upregulated in the PF of each of the four *Strongyloides* species, indicating some level of divergence between the molecular tool kits that each species uses in parasitism or other features associated with the parasitic life cycle stage. Between 25–305 gene families uniquely upregulated in the PF of one species, but not the other three species, were identified (Supplementary Tables S3–S6). The largest number of gene families and genes they comprise, was identified for *S*. *venezuelensis* (327 upregulated genes in 305 gene families). Most strikingly, genes encoding cytochrome P450 proteins were distinctly upregulated in the PF transcriptome of *S*. *venezuelensis*. Specifically, seven genes (of 13 predicted in the genome) predicted to encode cytochrome P450 family 4 subfamily V member 2 (4V2) proteins and 12 genes (of 34 genes in the genome) encoding cytochrome P450 18a1 subfamily of proteins were upregulated in the *S*. *venezuelensis* PF transcriptome, but were not upregulated in the PF transcriptomes of *S*. *papillosus*, *S*. *stercoralis*, and *S*. *ratti* (with the exception of one cytochrome P450 18a1 gene, of 24 predicted in the genome, upregulated in the PF of *S*. *papillosus*). Cytochrome P450s are a superfamily of heme proteins. Enrichment of the Molecular Function GO terms for electron carrier activity (GO0009055), heme binding (GO:0020037) and iron ion binding (GO:005506) exclusively in the PF (*cf*. FLF) transcriptome of *S*. *venezuelensis* but not the PF of the three other *Strongyloides* species further support a unique role of cytochrome P450s in the *S*. *venezuelensis* PF stage (Fig. 2).

Within the *Strongyloides* genus, closely related species have adapted and specialised to parasitise different host animals. Two species - *S*. *venezuelensis* and *S*. *ratti* - both infect rodents, including natural infection of brown rats (*Rattus norvegicus*) and experimental infection of mice under laboratory conditions. *S*. *venezuelensis* and *S*. *ratti* belong to different phylogenetic subclades and are likely to have evolved parasitism of rats independently^18^. Genes upregulated in the PF of *S*. *venezuelensis* and *S*. *ratti*, but not *S*. *papillosus* and *S*. *stercoralis* may therefore represent gene families that are specifically important for infecting rodent hosts e.g. genes coding for proteins with a role in avoiding or manipulating the rodent immune response. Interestingly, from our comparative analysis of the PF and FLF transcriptomes of all four *Strongyloides* species, we found a small number of gene families that were specifically upregulated in the *Strongyloides* rodent-parasites (Supplementary Table S14). In total, we identified 29 gene families comprising 32–34 genes upregulated in *S*. *venezuelensis* and *S*. *ratti* PF (*cf*. FLF), but not upregulated in the PF of *S*. *papillosus* and *S*. *stercoralis*, with each gene family comprising 1–3 upregulated genes per species. Based on previous analyses, most of these genes formed orthofamilies with genes from *S*. *papillosus* and *S*. *stercoralis*^3^ i.e. where orthologous genes were not differentially expressed between the PF and FLF stages. In *S*. *venezuelensis* and *S*. *ratti*, the fold change difference in expression levels between PF and FLF stages, although significant, was relatively low (*S*. *venezuelensis*: 3.03 ± 0.25, *S*. *ratti*: 2.50 ± 0.10). It is therefore possible that the gene families commonly upregulated in *S*. *venezuelensis* and *S*. *ratti* (but not *S*. *papillosus* and *S*. *stercoralis*) may represent sampling differences rather than true features of the PF transcriptome associated with *Strongyloides* rodent parasitism. To include gene families that may not be recognised or have a predicted function, we compared the orthofamilies (from Hunt *et al*.^3^) that were uniquely upregulated in the PF (*cf*. FLF) in pairwise species combinations. The number of orthofamilies uniquely upregulated in one species compared to the three other species ranged between 165–459 in PF and 146–529 in FLF (Supplementary Fig. S2). Species within each subclade were most similar to one another, with 60 orthofamilies specifically upregulated in the *S*. *venezuelensis-S*. *papillosus* subclade and 96 orthofamilies specifically upregulated in the *S*. *stercoralis-S*. *ratti* subclade. However, of the other pairwise species comparisons, *S*. *venezulensis* and *S*. *ratti* shared 50 orthofamiles upregulated in the PF, more than other pairwise comparisons (excluding subclade species pairs) which ranged between 9 and 39 orthofamilies. This suggests that the *S*. *venezulensis* and *S*. *ratti* PF transcriptomes are more similar to one another, outside of evolutionary subclades, and this may represent their ability to parasitise a common rodent host.

## Discussion

Comparative analysis of the transcriptomes of four *Strongyloides* species has identified key gene families with a putative role in parasitism by *Strongyloides* species, including gene families likely to have a role in parasitism at the genus (e.g. WAGO-like), subclade (e.g. SPOP-like), and species (e.g. cytochrome p450) level. In particular, this study has identified WAGO-like Argonautes associated with a parasitic (compared to free-living) life cycle stage. Studies on the small RNAs pathways associated with a role in nematode parasitism have predominantly focused on the microRNA pathway^19^, which is not mediated by WAGOs. The WAGO-like coding genes upregulated in *Strongyloides* PF may therefore represent another pathway involved in parasitism, for example, involving the secretion of WAGO-mediated small RNAs into the host to manipulate host genes expression. Alternatively, this expression pattern could be due to an upregulation of WAGO-like Argonautes in the matured ovaries or eggs of gravid parasitic, compared with eggs that have been released and the matured ovaries and eggs inside free-living adult females. Little is known about differences that exist between the eggs inside parasitic versus free-living females. In the *Strongyloides* life cycle, PF reproduce by mitotic parthenogenesis and FLF reproduce sexually^1,20^ and the differential expression of the WAGO-like Argonaute may therefore be associated with a role in parthenogenic reproduction.

The comparison of transcriptomic data for four *Strongyloides* species presented here enables inferences to be made about the divergent evolution of parasitism of two distinct phylogenetic *Strongyloides* subclades (*S*. *venezuelensis- S*. *papillosus* and *S*. *ratti-S*. *stercoralis*). Upregulation of genes in the PF that are related to ubiquitation (i.e. SPOP-like and F-box-coding genes), coupled with an expansion of the SPOP-like gene family specifically in *S*. *venezuelensis* and *S*. *papillosus* suggests a putative role of the Ubiquitation Proteasome Pathway (UPP) in parasitism or features associated with the parasitic life cycle stage in this subclade. However, because the UPP is central to the regulation of many diverse cellular processes^21^, the exact role, if any, of UPP in parasitism or features associated with the parasitic life cycle stage of the *S*. *venezuelensis-S*. *papillosus* subclade is not clear and remains to be investigated.

*Strongyloides* species share many similarities across their life cycle, for example, they all have a parasitic stage inhabiting the mucosa of the small intestine of their host and a free-living stage outside of the host. All known *Strongyloides* species parasitise tetrapod (mainly mammalians) hosts, but the hosts vary between *Strongyloides* species. We have identified genes coding for protein families that are uniquely upregulated in the PF of each species, which may represent adaptation to parasitising distinctly different hosts. It should also be noted that data for *S*. *venezuelensis* and *S*. *ratti* used in this analysis are from PFs raised in their natural rodent host, compared to *S*. *papillosus* and *S*. *stercoralis* PFs which were raised in permissive hosts (rabbits and gerbils, respectively). It is therefore possible that genes upregulated in the PF of *S*. *papillosus* and *S*. *stercoralis* may also represent a host-parasite interaction that has not co-evolved outside of laboratory conditions. Although *S*. *venezuelensis* and *S*. *ratti* both parasitise rodent hosts we found few gene families that are specifically upregulated in the PF of these two species, but not *S*. *papillosus* and *S*. *stercoralis*. Together, this suggests that *S*. *venezuelensis* and *S*. *ratti* have either evolved different molecular strategies to parasitise their rodent hosts, (represented by gene families that are uniquely upregulated in their respective PF transcriptomes) or they use similar genes and the proteins to infect rodents, but these are regulated at the post-transcriptional or post-translational level. Together, our results identify key gene families with a putative role in *Strongyloides* parasitism or features of the parasitic life cycle stage, and deepen our understanding of parasitism evolution among *Strongyloides* species.

## Methods

### Collection of *S. venezuelensis* samples and RNAseq

*S*. *venezuelensis* HH1 is used in this study, which have been maintained in the Parasitology laboratory at the University of Miyazaki. In brief, *S*. *venezuelensis* have been maintained in male Wistar rats by serial infection using subcutaneous injection of infective larvae prepared by faecal culture using filter paper^9^. Parasitic females were isolated from rat small intestine at 8 days post-infection (d.p.i). Faeces (approx. 1 g) collected from infected rats at 12 d.p.i were cultured on a 2% (w/v) agar plate at 25 °C for 3 days and FLF nematodes were collected. Twenty PF or 8–16 FLF were transferred to individual tubes containing 150 µL TRI reagent (Life Technology) and total RNA was extracted according to the standard procedures (Life Technology). RNA was then amplified using the SMARter Ultra Low amplification kit (Clontech) and sequencing libraries were constructed using the TruSeq Sample Prep kit according to the manufacturer’s recommended protocols (Illumina). The libraries were sequenced for 151-bp paired-ends on an Illumina MiSeq sequencer with MiSeq Reagent Kit v2 (300 cycles) using the standard protocol (Illumina) to obtain >15 million pair-end reads for each sample. RNAseq experiments were conducted in triplicate (biologically). Reads have been submitted to the DNA Data Bank of Japan (DDBJ) under BioSample accession numbers SAMD00096905-SAMD00096910.

All the animal experiments were performed in accordance with the procedures approved by the Animal Experiment Committee of the University of Miyazaki under an approval no. 2009-506-6.

### Expression data analysis

*S*. *venezuelensis* RNAseq reads (this study) and *S*. *papillosus* data (downloaded from the European Nucleotide archive (accession no. PRJEB14543)) were aligned to the genome references (v2.0 and v2.1, respectively) using TopHat2^22^ using the same parameters as Hunt *et al*.^3^ for *S*. *stercoralis* and *S*. *ratti* (parameters: –a 6 –i 10 –I 20000–microexon_search–min-segment-intron 10–max-segment-intron 20000). Read counts per gene and fragments Per Kilobase of transcript per Million mapped reads (FPKM) were calculated using HT-seq.^23^ and cufflinks^24^ packages, respectively. Differential expression of genes was determined by edgeR^25^ analysis using R version 3.3.3. Common dispersion values 0.07379822, 0.1692273, 0.006412771 and 0.007270067 for *S*. *venezuelensis*, *S*. *papillosus*, *S*. *ratti* and *S*. *stercoralis*, respectively, were estimated for all genes using the edgeR package^25^ n.b. *S*. *papillosus* has a higher dispersal rate compared to the three other *Strongloides* species (Supplementary Fig. S1). Genes were considered differentially expressed if they had a fold-change of at least 2 and a FDR adjusted p-value < 0.05. Genes were categorised into the gene families based on the predicted protein function, as described by Hunt *et al*.^3^. For *S*. *ratti* and *S*. *stercoralis*, previously analysed expression data for PF and FLFs was downloaded from ArrayExpress (accession nos. E-ERAD-151 and E-ERAD-92, and E-MTAB-1164, respectively^3,26^). The same methods and parameters for transcriptome analysis were used for all four *Strongyloides* species. RNAseq data of *S*. *venezuelensis* other stages were retrieved from DDBJ under BioProject accession number PRJDB3457.

### Gene Ontology (GO) analysis

Enriched GO terms for *S*. *venezuelensis* and *S*. *papillosus* PF and FLF life cycle stages were established using the TopGO^27^ package in R version 3.3.3. For comparative analyses with *S*. *ratti* and *S*. *stercoralis*, GO term enrichment analysis from Hunt *et al*.^3^ were used, which were analysed using the same method as described here for *S*. *venezuelensis* and *S*. *papillosus*.

### Identification of genes encoding protein families

*Speckle-type POZ-like* (*SPOP-like*) *genes:* the SPOP-like gene family was identified based on the predicted protein function as described by Hunt *et al*.^3^. Further analysis of protein domain predictions was carried out using InterProScan searches for BTB/POZ InterProScan domain (IPR000210) and MATH/ TRAF domain (IPR000210).

*Argonaute genes:* Genes predicted to encode Argonaute proteins were identified by the presence of a PIWI domain (IPR003165) characteristic of Argonaute proteins and prediction of either a PAZ (IPR003100) or ribonuclease h-like domain (IPR012337).

### Phylogenetic analysis

Amino acid sequences of SPOP-like and Argonautes were individually aligned using MUSCLE^28^ and phylogenetic trees were produced using RAxML with –m PROTGAMMAAUTO and 100 bootstraps^29^. For both phylogenies, the GAMMA ML model was selected as the best fit to the data. For the construction of phylogenetic trees, genes from 14 nematode species were used to represent species across multiple evolutionary clades. These species are *S.* *venezuelensis*, *S*. *papillosus*, *S*. *stercoralis*, *S*. *ratti*, *P*. *trichosuri*, *Rhabditophanes* sp., *Caenorhabditis elegans*, *Necator americanus*, *Meloidogyne hapla*, *Trichinella spiralis*, *Ascaris suum*, *Brugia malayi*, *Bursaphelenchus xylophilus* and *Trichuris muris*.

### Nomenclature

#### Gene families

Families of genes grouped according to the predicted function of the protein they code for. The same parameters were used for the annotation of protein function for the predicted genes for each of the four *Strongyloides* species used in this analysis, and UniProt’s naming guidelines were used to assign protein names (http://www.uniprot.org/docs/nameprot), as described by Hunt *et al*.^3^.

#### Orthofamilies

Families of orthologous genes based on previous analysis^3^ using EnsemblCompara^30^.

## Electronic supplementary material

Supplementary Fig. S1 to S5

Supplementary Tables S1 to S14

## References

[1] Viney ME (1994). A genetic analysis of reproduction in Strongyloides ratti. Parasitology.

[2] Schad, G. In *Strongyloidiasis a major roundworm infection of man* (ed. Grove, D.) 85–104 (Taylor and Francis, 1989).

[3] Hunt VL (2016). The genomic basis of parasitism in the Strongyloides clade of nematodes. Nat. Genet..

[4] Hunt VL, Tsai IJ, Selkirk ME, Viney M (2017). The genome of Strongyloides spp. gives insights into protein families with a putative role in nematode parasitism. Parasitology.

[5] Williamson AL (2006). Ancylostoma caninum MTP-1, an astacin-like metalloprotease secreted by infective hookworm larvae, is involved in tissue migration. Infect. Immun..

[6] Lee DL (1996). Why do some nematode parasites of the alimentary tract secrete acetylcholinesterase?. Int. J. Parasitol..

[7] Del Valle A, Jones BF, Harrison LM, Chadderdon RC, Cappello M (2003). Isolation and molecular cloning of a secreted hookworm platelet inhibitor from adult Ancylostoma caninum. Mol. Biochem. Parasitol..

[8] Nemetschke L, Eberhardt AG, Hertzberg H, Streit A (2010). Genetics, chromatin diminution, and sex chromosome evolution in the parasitic nematode genus Strongyloides. Curr. Biol..

[9] Hino A (2014). Karyotype and reproduction mode of the rodent parasite Strongyloides venezuelensis. Parasitology.

[10] Kulkarni A, Dyka A, Nemetschke L, Grant WN, Streit A (2013). Parastrongyloides trichosuri suggests that XX/XO sex determination is ancestral in Strongyloididae (Nematoda). Parasitology.

[11] Baskaran P, Jaleta TG, Streit A, Rödelsperger C (2017). Duplications and Positive Selection Drive the Evolution of Parasitism-Associated Gene Families in the Nematode Strongyloides papillosus. Genome Biol. Evol..

[12] Das PP (2008). Piwi and piRNAs Act Upstream of an Endogenous siRNA Pathway to Suppress Tc3 Transposon Mobility in the Caenorhabditis elegans Germline. Mol. Cell.

[13] Billi, A. C., Fischer, S. E. & Kim, J. K. Endogenous RNAi pathways in C. elegans. *WormBook* 1–49, 10.1895/wormbook.1.170.1 (2014). 10.1895/wormbook.1.170.1 PMC4781133 24816713

[14] Buck AH, Blaxter M (2013). Functional diversification of Argonautes in nematodes: an expanding universe. Biochem. Soc. Trans..

[15] Holz A, Streit A (2017). Gain and Loss ofSmall RNA Classes—Characterization of Small RNAs in the Parasitic Nematode Family Strongyloididae. Genome Biol. Evol..

[16] Bardwell VJ, Treisman R (1994). The POZ domain: A conserved protein-protein interaction motif. Genes Dev..

[17] Xu L (2003). BTB proteins are substrate-specific adaptors in an SCF-like modular ubiquitin ligase containing CUL-3. Nature.

[18] Viney M, Kikuchi T (2017). Strongyloides ratti and S. venezuelensis – rodent models of Strongyloides infection. Parasitology.

[19] Buck AH (2014). Exosomes secreted by nematode parasites transfer small RNAs to mammalian cells and modulate innate immunity. Nat. Commun..

[20] Viney ME, Matthews BE, Walliker D (1993). Mating in the nematode parasite Strongyloides ratti: proof of genetic exchange. Proc. Biol. Sci..

[21] Ciechanover A (1998). The ubiquitin-proteasome pathway: On protein death and cell life. EMBO Journal.

[22] Kim D (2013). TopHat2: accurate alignment of transcriptomes in the presence of insertions, deletions and gene fusions. Genome Biol..

[23] Anders S, Pyl PT, Huber W (2015). HTSeq-A Python framework to work with high-throughput sequencing data. Bioinformatics.

[24] Trapnell C (2012). Differential gene and transcript expression analysis of RNA-seq experiments with TopHat and Cufflinks. Nat. Protoc..

[25] Robinson MD, McCarthy DJ, Smyth G (2010). K. edgeR: a Bioconductor package for differential expression analysis of digital gene expression data. Bioinformatics.

[26] Stoltzfus JD, Minot S, Berriman M, Nolan TJ, Lok JB (2012). RNAseq analysis of the parasitic nematode Strongyloides stercoralis reveals divergent regulation of canonical dauer pathways. PLoS Negl. Trop. Dis..

[27] Alexa, A. & Rahnenführer, J. Gene set enrichment analysis with topGO. *Bioconductor Improv*. 27 at http://scholar.google.com/scholar?hl=en&btnG=Search&q=intitle:Gene+set+enrichment+analysis+with+topGO#0%5Cnftp://mirrors.nic.funet.fi/bioconductor.org/2.7/bioc/vignettes/topGO/inst/doc/topGO.pdf (2007).

[28] Edgar RC (2004). MUSCLE: multiple sequence alignment with high accuracy and high throughput. Nucleic Acids Res..

[29] Stamatakis A (2014). RAxML version 8: a tool for phylogenetic analysis and post-analysis of large phylogenies. Bioinformatics.

[30] Vilella AJ (2009). EnsemblCompara GeneTrees: Complete, duplication-aware phylogenetic trees in vertebrates. Genome Res..

[31] Supek, F., Bošnjak, M., Škunca, N. & Šmuc, T. Revigo summarizes and visualizes long lists of gene ontology terms. *PLoS One* **6**, (2011). 10.1371/journal.pone.0021800 PMC3138752 21789182

[32] Blaxter ML (1998). A molecular evolutionary framework for the phylum Nematoda. Nature.

