# Supplementary material for: Comparative transcriptomics gives insights into the evolution of parasitism in Strongyloides nematodes at the genus, subclade and species level
Source: Sci Rep. 2018 Mar 26;8:5192. doi: 10.1038/s41598-018-23514-z (PMC5979966; doi:10.1038/s41598-018-23514-z)
Supplement: Supplementary file 1 — Supplementary Fig. S1 to S5 [file 41598_2018_23514_MOESM1_ESM.pdf]

Supplementary Materials for

Comparative transcriptomics gives insights into the evolution of parasitism in *Strongyloides* nematodes at the genus, subclade and species level

Vicky L. Hunt[1], Akina Hino[1,2], Akemi Yoshida[3], Taisei Kikuchi[1*]

[1] Parasitology, Faculty of Medicine, University of Miyazaki, Miyazaki, 889-1692 Japan

[2] Department of Environmental Parasitology, Graduate School of Medical and Dental Sciences, Tokyo Medical and Dental University, 1-5-45 Yushima, Bunkyo-ku, Tokyo, 113-8510, Japan.

[3] Genomics and Bioenvironmental Science, Frontier Science Research Center, University of Miyazaki, 889-1692 Japan

*Corresponding author

email: taisei_kikuchi@med.miyazaki-u.ac.jp

tel: +81-985850990, fax: +81-985843887

This PDF file includes:

Supplementary Figs. S1 to S7

Other Supplementary Materials for this manuscript includes the following:

Supplementary Tables S1 to S14 (an excel file)

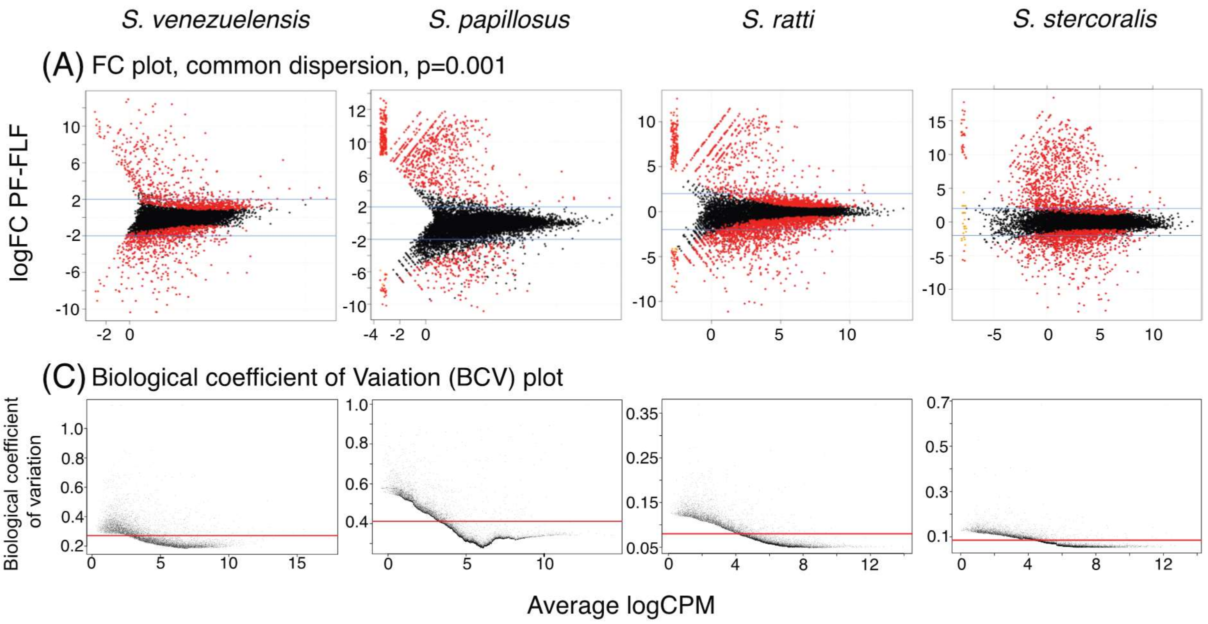

Supplementary Fig. S1. MA plots (A) and biological coefficient of variation (BCV) plots (B) generated from edgeR analysis of PF and FLF transcriptomic data for *S. venezuelensis, S. papillosus, S. ratti* and *S. stercoralis*. (A) Each gene is represented as a single data point and is coloured to represent either no differential expression (black), significantly upregulated when the or p=0.001 (red) and p=0.05 (orange). Genes were only considered to be significantly upregulated if they were significantly different at p=0.05 level and had a fold change (FC) greater than 2, indicated by the blue lines. CPM = count per million mapped reads. Note, the plots for *S. ratti* and *S. stercoralis* are from the data presented in Hunt *et al* (2016)[3]. (B) Each data point represents a gene; the red line represents the common dispersion, as estimated by edgeR[25].

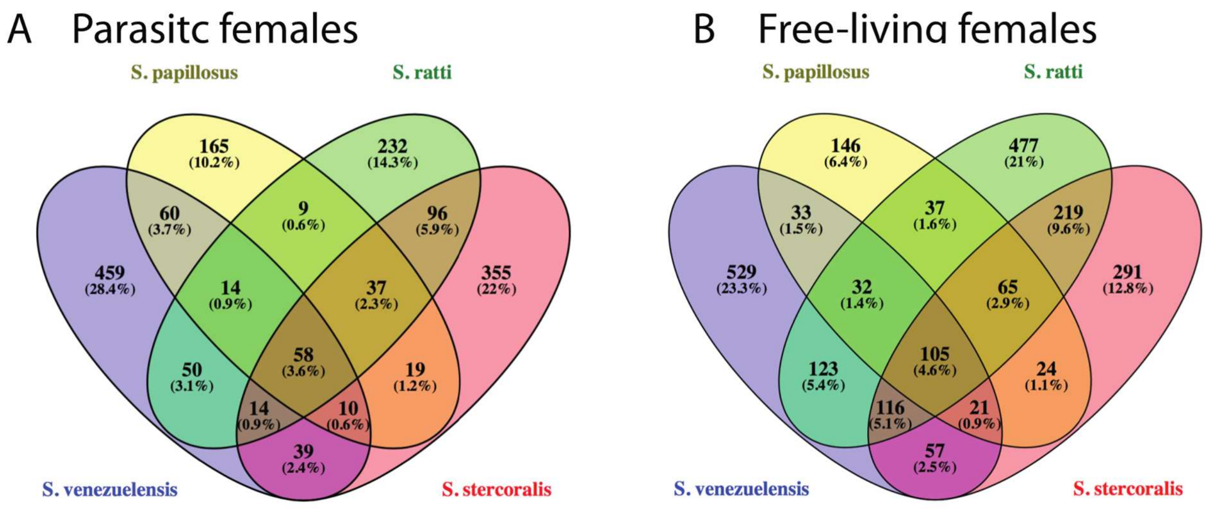

Supplementary Fig. S2. Differentially expressed orthofamilies. A summary of the number of orthofamilies that are commonly and uncommonly upregulated across four *Strongyloides* species (*S. venezuelensis* (blue), *S. papillosus* (yellow), *S. ratti* (green) and *S. stercoralis* (red)) in (A) parasitic adult females and (B) free-living adult female stages of the *Strongyloides* life cycle. An orthofamily was considered to be upregulated in the PF or FLF of a *Strongyloides* species if at least one gene from the orthofamily was significantly upregulated compared to the other life cycle stage. Orthofamilies are determined using EnsemblCompara by Hunt *et al* (2016)[3]. Venn diagrams were generated using Venny version 2.1.0[33].

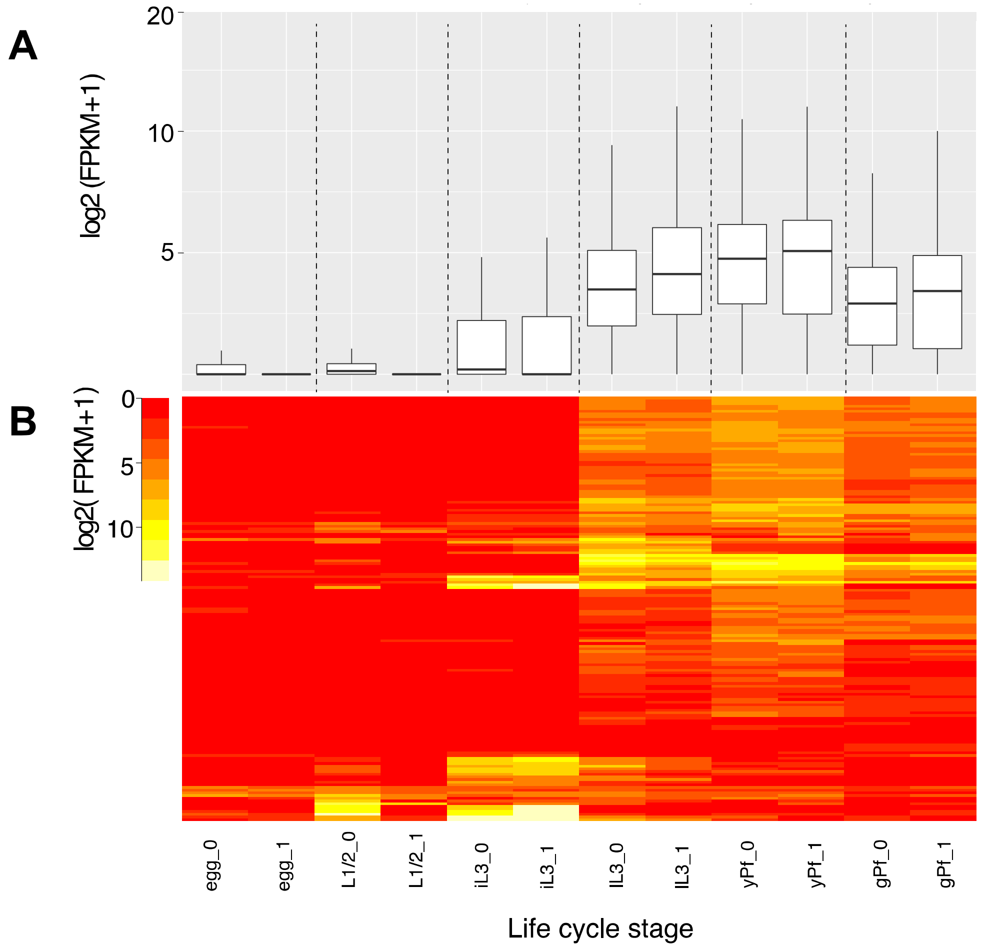

Supplementary Fig. S3. Expression of SCP/TAPS-coding genes in *S. venezuelensis.* (A) Boxplots of the log2 FPKM values from RNAseq data for all 159 genes predicted to code for a SCP/TAPS. RNAseq data is shown for six stages of the *S. venezuelensis* life cycle including eggs, first/second-stage larvae (L1/2), infective third-stage larvae (iL3), infective larvae isolated from the host lung (lL3), young PF (yPF) and gravid PF (gPF). Outlier data points are not shown. (B) Heatmap expression profile for all SCP/TAPS-coding genes, calculated from log2 FPKM values from RNAseq data. Each life cycle stage has two biological replicates. FPKM = Fragments Per Kilobase per Million mapped reads. All FPKM values have been transformed by addition of 1 and log2.

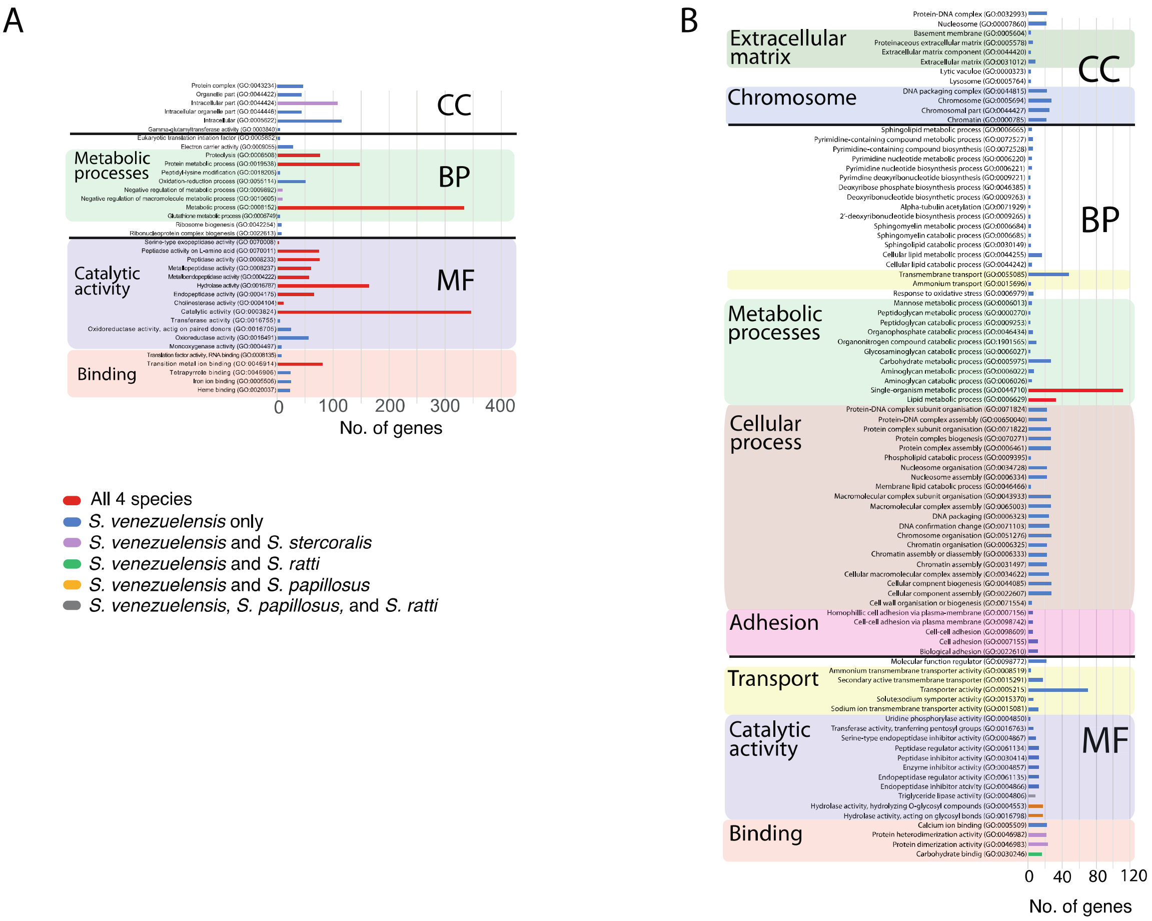

Supplementary Fig. S4. Gene Ontology (GO) terms that are overrepresented in the upregulated transcriptome of PF (A) and FLF (B) stages of the *S. venezuelensis* life cycle. GO terms commonly overrepresented in the same life cycle stage of other *Strongyloides* species are highlighted by colour including GO terms upregulated in all four *Strongyloides* species (red), only upregulated in *S. venezuelensis* (blue), upregulated only in *S. venezuelensis* and *S. stercoralis* (mauve), *S. venezuelensis* and *S. ratti* (green), *S. venezuelensis* and *S. papillosus* (orange), *S. venezuelensis*, *S. papillosus* and *S. ratti* (grey).

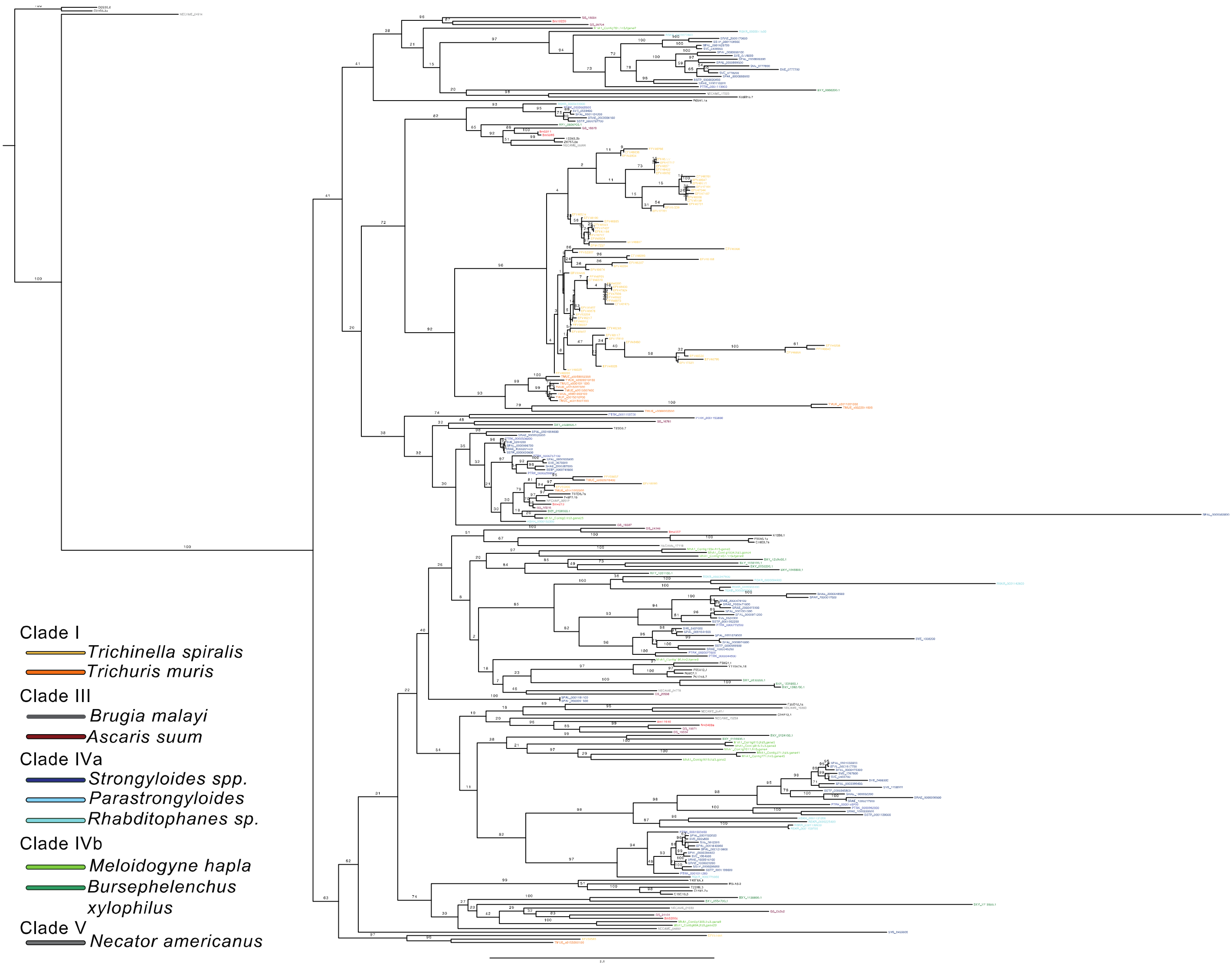

Supplementary Fig. S5. Phylogeny of presumed Argonaute genes, including gene names. Amino acid sequences for predicted Argonaute-coding genes from 14 nematodes species, spanning five evolutionary clades (as defined by Blaxter *et al*[32]), were used to construct a phylogeny. Branch support values are from 100 bootstraps. Genes coding for predicted Argonaute proteins are coloured according to species except for the four *Strongyloides* species (*S. venezuelensis, S. papillosus, S. ratti* and *S. stercoralis*) which are all colour-coded dark blue. Genes belonging to each species are colour-coded with similar colours according to their evolutionary clade (as defined by Blaxter *et al*[32]). The protein names of *C. elegans* Argonautes are shown directly on the phylogeny in black text. A simplified version of this phylogenetic tree is shown in **Figure 3**.

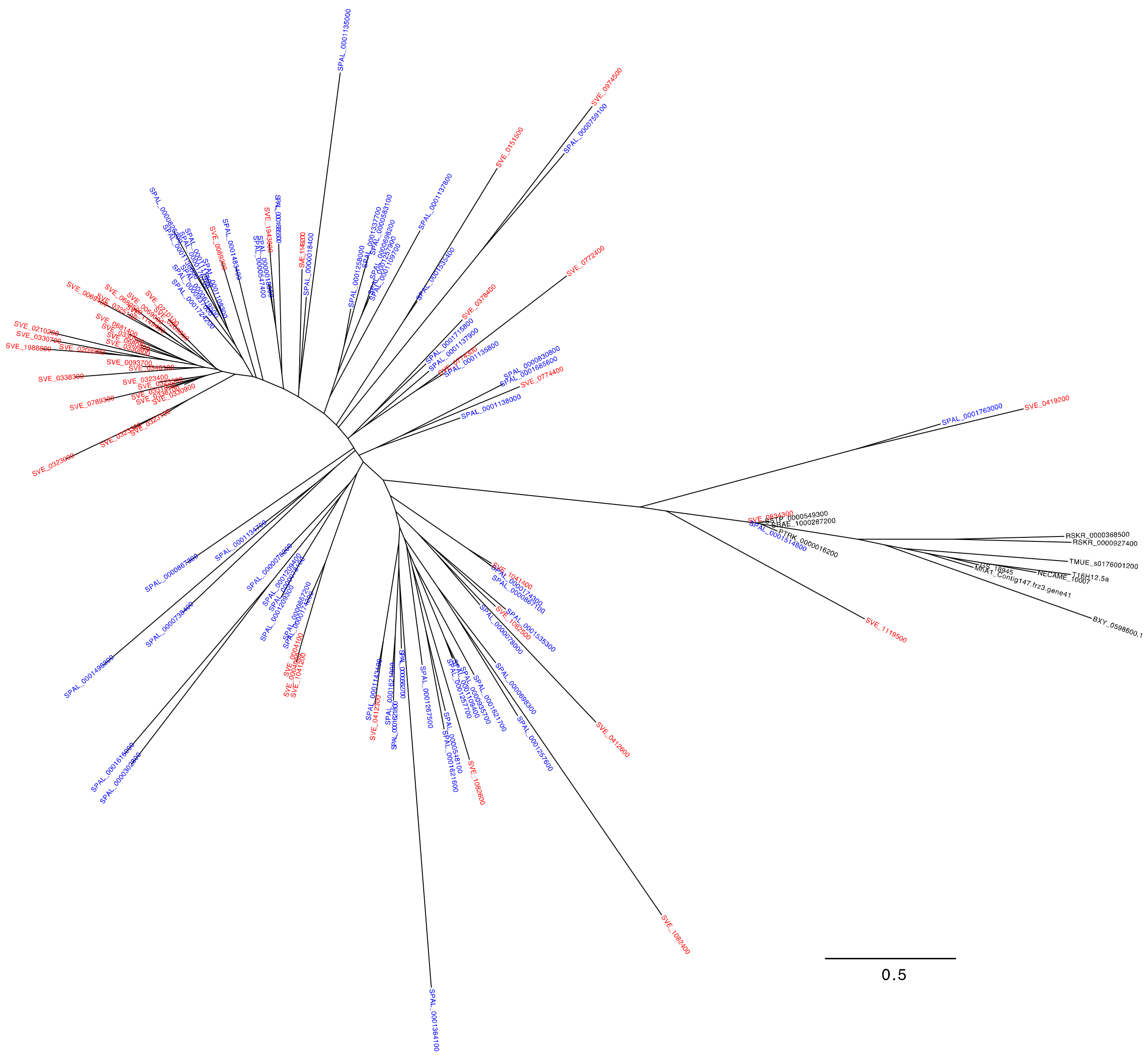

Supplementary Fig. S6. Phylogeny of speckle-type POZ-like (SPOP-like) including gene names. Amino acid sequences predicted SPOP-like genes from 14 nematodes species, spanning five evolutionary clades (as defined by Blaxter *et al*[32]), were used to construct a phylogeny. SPOP-like genes are highlighted for *S. venezuelensis* (red) and *S. papillosus* (blue); genes for all other species are shown in black. Phylogeny of speckle-type POZ-like (SPOP-like) with gene names. A simplified version of this phylogenetic tree is shown in **Figure 5**.
